# Ovarian Mixed Malignant Brenner-Mucinous Tumor with Signet Ring Cells

**DOI:** 10.1155/2020/2134546

**Published:** 2020-03-26

**Authors:** Baharak Khadang, Atilla Omeroglu

**Affiliations:** Department of Pathology, McGill University, Montreal, Canada

## Abstract

Mucinous carcinomas with signet ring cells in the ovary, particularly those composed predominantly of signet ring cells, are extremely rare, and in vast majority of cases, they represent metastasis from another site such as the stomach, appendix, pancreaticobiliary tract, bladder, and breast (Hristov et al., 2007, Kiyokawa et al., 2006, Vang et al., 2006, Young, 2006). Malignant Brenner tumor is also rare comprising less than 0.5% of ovarian carcinoma. Although mixed Brenner-Mucinous tumors are relatively common, the combination of a primary ovarian signet ring carcinoma with a malignant Brenner tumor is unique and to the best of our knowledge not previously reported in the literature.

## 1. Case Report

A 78-year-old woman was referred to McGill University Health Center with a large abdominal mass. She had a computed tomography (CT) scan of her abdomen and pelvis, which showed a mass measuring 19 × 18.4 × 15.5 cm with heterogenous enhancement and bilateral mild hydronephrosis. There was no sign of invasion into adjacent bowel and vessels and no obvious abdominal or pelvic lymphadenopathy. She had a past surgical history of bilateral tubal ligation and an appendectomy, cholecystectomy, right total hip replacement, and tonsillectomy. She was otherwise healthy. Her tumor markers were almost within normal limits. CA125 was 37, slightly elevated, and CA19.9 was 20, and CEA was elevated to 7. The patient underwent total abdominal hysterectomy with bilateral salpingo-oophorectomy and partial omentectomy.

The removed right ovary exhibited a sizeable mass, measuring 24 × 20 × 11 cm and weighing 2930 grams. The external surface had an intact and smooth capsule. On sectioning, the mass contained a multiloculated cystic component filled with abundant gelatinous mucinous substance.

**Figure 1:**
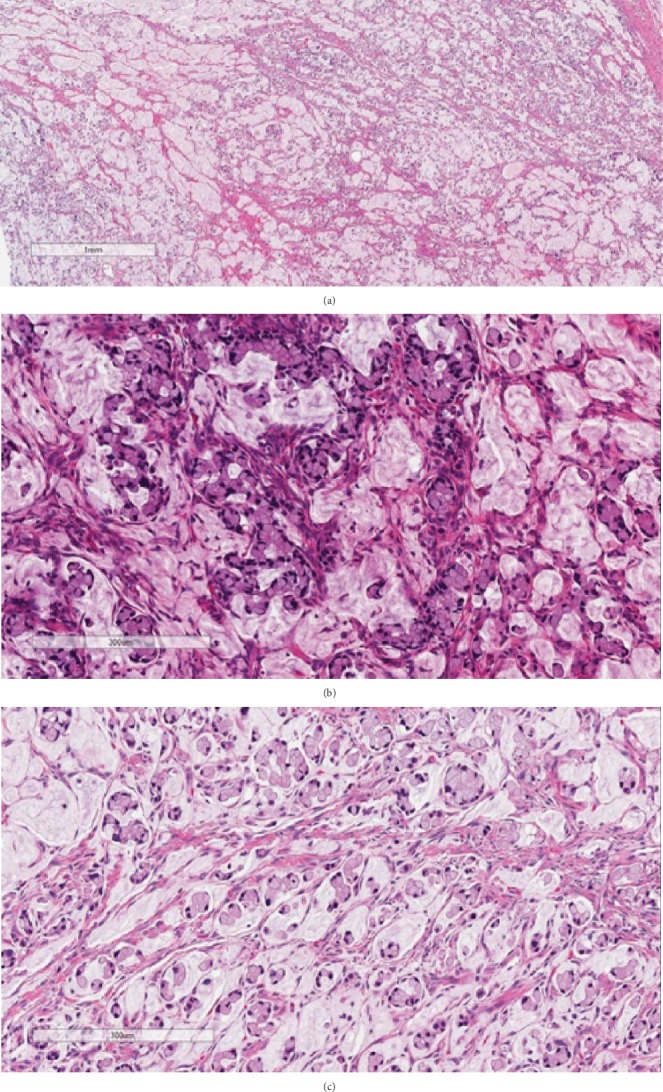
The tumor predominantly consists of signet ring cells floating in mucinous substance (hematoxylin and eosin staining; magnification: (a) ×40 and (b, c) ×200).

The left ovary, uterus, cervix, and omentum appeared to be normal on macroscopic examination. Histologically, most of the mass was composed of single or clusters of signet ring cells arranged in small groups or individual cells admixed with abundant extracellular mucin (Figure 1). The stroma was mostly composed of delicate fibrous bands in between the signet ring cells. There was no evidence of lymphovascular invasion, nodular growth pattern, or tumor cells on the ovarian surface.

The tumor cells exhibited diffuse positivity for cytokeratins CK7 and 20 and also for CDX-2. However, there was no expression of ER, PR, HER-2, and GATA-3. PAX-8 was weakly positive in focal areas.

**Figure 2:**
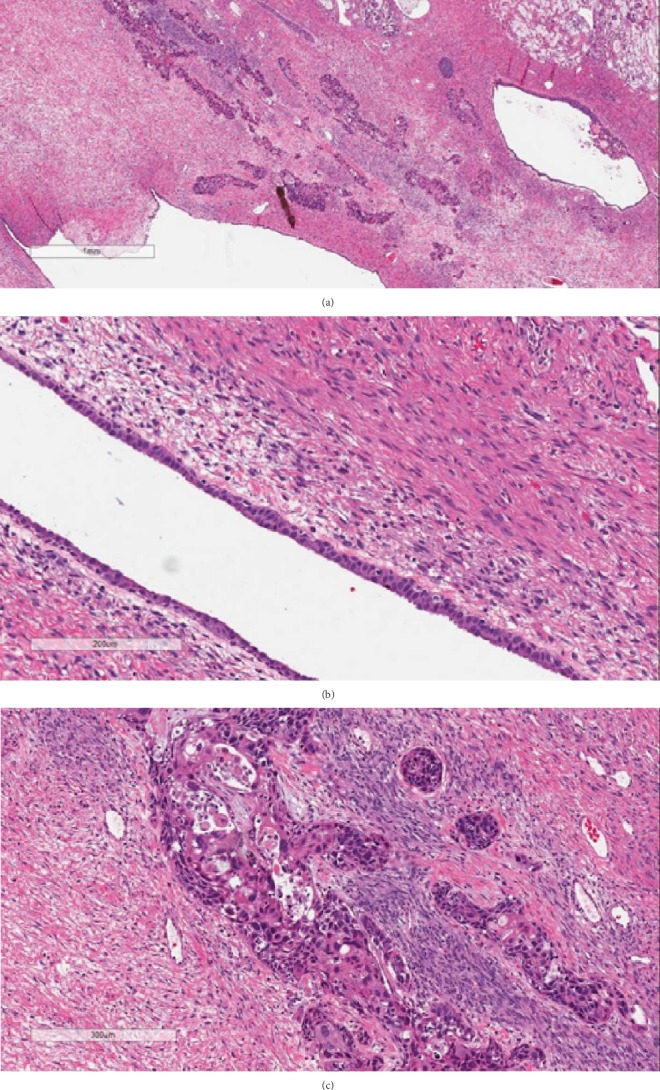
(a) Brenner tumor component from low power (×20 magnification; H&E stain). (b) Benign Brenner tumor lined with transitional epithelium in a fibrous stroma (×200 magnification; H&E stain). (c) Malignant Brenner tumor invading adjacent stroma (×200 magnification; H&E stain).

Furthermore, extensive sampling of the mass revealed a focus of cystic structures lined by benign transitional like epithelium and a focal area showing highly atypical, pleomorphic transitional to squamoid cells with brisk mitotic activity invading adjacent stroma. This was consistent with presence of both benign and malignant components of a Brenner tumor (Figure 2). The Brenner tumor composed only a small proportion of the whole mass, measuring 1.2 cm in largest dimension. The Brenner tumor was positive for GATA-3, P63, and CK 7 and negative for CK 20.

Postoperative computed topography (CT) from the chest, abdomen, and pelvis revealed no residual malignancy and no alternative primary site. The extensive upper and lower gastrointestinal workups did not show any primary tumor as well. The cytology of the peritoneal fluid was negative for malignant cells. Based on these clinicoradiological and histopathological findings, this case was diagnosed as primary signet ring cell carcinoma of the ovary with benign and malignant Brenner tumor components. One-year follow-up after the surgery revealed no evidence of recurrence. The patient remains alive and well.

## 2. Discussion

The presence of signet ring cells in an ovarian mucinous carcinoma or a predominant signet ring cell component is generally highly suggestive of a metastatic neoplasm, most likely from gastrointestinal tract referred to as Krukenberg tumor and less common from appendix, pancreaticobiliary tract, bladder, and breast [1–5]. In addition to signet ring cells, bilaterality, nodular growth, small size, surface implants, stromal invasion, prominent histological variation between different areas, tumor cells floating in mucin, extraovarian spread, and lymphovascular invasion, especially at the hilum, are features mostly suggestive of a metastatic mucinous neoplasm [6, 7]. The distinction between primary signet ring cell carcinoma of ovary and metastasis may be very challenging though. So far, only rare cases of primary signet ring cell carcinoma of the ovary have been reported. In 1968, Joshi made an extensive review of “Primary Krukenberg Tumors of Ovary” [8]. In 2001, Che and colleagues reported the presence of signet ring cells in surface epithelial carcinomas of the ovary and their occurrence in serous and endometrioid carcinomas [9].

In 2007, Vang et al. reported 3 cases of mucinous carcinoma with signet ring cells associated with dermoid cysts [10]. Most of the studies so far report a primary ovarian mucinous neoplasm with focal or extensive signet ring cell features rather than a predominant component of signet ring cells. The current exploration of this topic continues, considering a noteworthy case report of ovarian signet ring cell carcinoma with predominantly signet ring cells by Kim et al. [11].

Some studies have reported signet ring cell carcinoma arising from a teratomatous component [12].

Similar to the study by Kim et al. [11], we believe that in the present case, the neoplasm is an ovarian primary. To the best of our knowledge, this is the first case in the literature with primary ovarian signet ring cell carcinoma and malignant Brenner tumor. Although the presence of signet ring cells is a key pathological characteristic favoring a metastasis from other primary sites, the present case was considered to be a primary neoplasm based on the following findings: unilaterality, large size, lack of surface involvement and implantation, and no extraovarian spread. In addition, the tumor displayed admixed components of benign and malignant Brenner tumor. Furthermore, extensive clinical investigation did not reveal an extraovarian primary. The derivation of ovarian mucinous tumors is not well established, and some studies suggest the origin of some of these tumors from teratomas [13–15]. However, it has been recently proposed that these tumors may originate from Brenner tumors, and an association of a mucinous tumor with a Brenner tumor has been frequently observed [16]. In a noteworthy study by Wang et al. [17], they demonstrated that in combined mucinous and Brenner tumors, there is a shared clonal relationship between the two different tumor components and suggested that some pure mucinous tumors may develop from a Brenner tumor. They speculated that the transitional epithelium of Brenner tumor undergoes cell lineage reprogramming to mucinous epithelium through metaplasia. The metaplastic epithelium proliferates and gives rise to a mucinous tumor [18]. The coexistence of Brenner tumor with signet ring cell carcinoma in our case concords with the abovementioned hypothesis and also the primary ovarian origin of signet ring cells.

Several primary ovarian mucinous neoplasms display intestinal differentiation and express CK20 and CDX-2, in addition to CK7 [19]. However, these tumors are mostly negative for ER and PR and variably express PAX-8. As the enteric markers are variably positive in metastatic mucinous tumors from the stomach, pancreatobiliary tract, appendix, and colorectum, immunohistochemistry may be overall not very helpful in distinguishing the primary or secondary origin of an ovarian signet ring cell carcinoma. Clinical and radiological correlation in these cases is of significant value to ensure correct diagnosis.

In our case, possible primary tumor in the female genital tract was excluded following the surgery. No other lesions in the gastrointestinal tract were identified on CT and upper and lower gastrointestinal endoscopy. The remaining small possibility of a small occult primary neoplasm in other organs, most likely the stomach and appendix, has been excluded so far in clinical follow-up. Taking into account the histopathological findings and clinical investigations, we render a diagnosis of primary ovarian signet ring cell carcinoma with associated malignant Brenner tumor.
